# Elevated Osteopontin Levels in Mild Cognitive Impairment and Alzheimer's Disease

**DOI:** 10.1155/2013/615745

**Published:** 2013-03-14

**Authors:** Yuan Sun, Xue Song Yin, Hong Guo, Rong Kun Han, Rui Dong He, Li Jun Chi

**Affiliations:** ^1^Department of Neurology, Bei Jing Daopei Hospital, Bei Jing 100749, China; ^2^Department of Emergency, The Fourth Affiliated Hospital of Harbin Medical University, Harbin 150001, China; ^3^Department of Paediatrics, Daqing Oilfied General Hospital, Daqing 163311, China; ^4^Department of Neurology, The First Affiliated Hospital of Harbin Medical University, Harbin 150001, China

## Abstract

Inflammatory mediators are closely associated with the pathogenesis of neurodegenerative changes in Alzheimer's disease (AD) and mild cognitive impairment (MCI). Osteopontin (OPN) is a proinflammatory cytokine that has been shown to play an important role in various neuroinflammatory diseases. However, the function of OPN in AD and MCI progression is not well defined. Cerebrospinal fluid (CSF) and plasma samples were obtained from 35 AD patients, 31 MCI patients, and 20 other noninflammatory neurologic diseases (OND). Concentrations of OPN in the CSF and plasma were determined by enzyme-linked immunosorbent assay. During a 3-year clinical followup, 13 MCI patients converted to AD (MCI converters), and 18 were clinically stable (MCI nonconverters). CSF OPN concentrations were significantly increased in AD and MCI converters compared to OND, and increased levels of OPN in AD were associated with MMSE score; OPN protein levels both in the CSF and plasma of newly diagnosed AD patients were higher than that of chronical patients. In MCI converters individuals tested longitudinally, both plasma and CSF OPN concentrations were significantly elevated when they received a diagnosis of AD during followup. Further wide-scale studies are necessary to confirm these results and to shed light on the etiopathogenic role of osteopontin in AD.

## 1. Introduction

Alzheimer's disease (AD) is a prominent neurodegenerative disorder characterized by chronically progressive global cognitive decline [1]. Mild cognitive impairment (MCI) has been recognized as intermediate between normal elderly cognition and dementia. A proportion of individuals with MCI may progress to AD and have typical biological changes of this kind of dementia [2]. To date, the underlying mechanisms involved in the degeneration of cerebral neurons and synapses in AD remain an enigma, but the theory about the involvement of inflammatory processes and immune dysregulation in their pathogenesis has been widely demonstrated. Neuropathological and neuroradiological studies have demonstrated that inflammatory changes in AD brains are a relatively early pathogenic event that precedes the process of neuropil destruction [3, 4]. In addition, studies have demonstrated that the brains and CFS of AD and MCI contained various proinflammatory substances, such as cytokines, acute phase proteins, and complement proteins [5–7], which have a fundamental role in inducing cognitive decline, memory loss, and dementia. It has been proposed that the autoimmune mechanism may be a trigger for AD, which was confirmed by the presence of autoantibodies and the apparently good outcome after immunotherapy as seen both in the animal model and in a few patients tested. Circulating autoantibodies against A*β* are several times higher in individuals with AD, and antibody titers correlate with cognitive dysfunction [8]. Active immunization and the use of monoclonal antibodies for passive vaccination have provided encouraging data in transgenic mouse models of AD [9].

Osteopontin (OPN), also called “early T cell-activation gene 1” [10], is a negatively charged acidic hydrophilic protein that is produced by various cell types and participates in diverse physiological and pathological processes, including bone mineralization, oxidative stress, remyelination, wound healing, inflammation, and immunity [11–13]. Studies have demonstrated marked upregulation of OPN in various inflammatory and autoimmune diseases. The expression of OPN was elevated in the brains of rats with experimental autoimmune encephalomyelitis (EAE) but not in brains of rats protected from EAE, and severity of EAE was significantly reduced in OPN deficient mice [14]. In concordance with those findings in animal models, OPN transcripts were frequently detected and were exclusive to the multiple sclerosis mRNA population, but not found in control brain mRNA [15]. In addition, the expression levels of OPN in plasma and tissues are also elevated in other several inflammatory or autoimmune disorders, such as rheumatoid arthritis, inflammatory bowel disease, systemic lupus erythematosus, and lymphoproliferation disease [16–19]. It has been well studied that interactions between OPN and its receptors (including *α*v*β*3, *α*5*β*1 and CD44) mediated survival, migration, and adhesion in many types of cells [20, 21]. As a proinflammatory mediator, OPN plays a role in the progression of chronic inflammatory and autoimmune diseases through various mechanisms, including involving in generation of Th1 and Th17 cells that are pathogenic T cells for various inflammatory diseases [22–24], inhibiting apoptosis of autoreactive immune cells and recruitment of leukocytes to sites of inflammation [21, 25].

Recently, the important role of OPN in AD has been investigated both in humans and animals model [26]. In AD brains, there was a significant 41% increase in the expression of OPN in pyramidal neurons compared with age-matched control brain, and there was a significant positive correlation between OPN staining intensity and amyloid-beta load [27]. By means of proteomic analysis of CSF samples, Simonsen and colleagues identified a phosphorylated C-terminal fragment of OPN that was increased in patients with MCI progressing to AD as compared to patients who remain stable over time and healthy controls [28]. Comi et al. demonstrated that OPN levels are increased in the CSF of AD subjects as compared to controls and its levels are more markedly raised in the early stages of the disease and correlate with cognitive decline [29]. In addition, upregulated OPN expression has also been demonstrated in A*β*PP/PS1 KI mice, an animal model of AD with severe pathological alterations [30]. Collectively, these findings strongly suggest the involvement of OPN in the development of AD. 

To further clarify the role of OPN in the progression of cognitive decline, we longitudinally assessed the OPN expression changes in the plasma and CSF in the same individuals. On the other hand, we transversely analyzed OPN levels in patients with MCI, newly diagnosed AD, and chronical AD, comparing the results with those obtained in the groups of healthy control and other noninflammatory neurologic diseases (OND).

## 2. Materials and Methods

### 2.1. Patients

Thirty-five patients affected by AD and Thirty-one patients with a diagnosis of MCI were selected for the study.  Table 1 lists the demographic data of the subjects, including gender, age, and the Mini-Mental State Examination Score (MMSE) [31], which is a general measure of cognitive performance.

The clinical diagnosis of AD was performed according to NINCDSADRDA work group criteria and DSM IV-R [32, 33]. The mean age of AD patients (16 males and 19 females) was 78.2 years (age range 58–80 years). All patients underwent complete medical and neurological evaluation, laboratory analysis, CT scan, or MRI to exclude reversible causes of dementia. Standard laboratory tests performed at the time of diagnosis included complete blood count, serum electrolytes, serum glucose, blood urea nitrogen, B12, folate, thyroid function tests, and serology for syphilis. Neuropsychological evaluation and psychometric assessment was performed with a Neuropsychological Battery including MMSE, Digit Span Forward and Backward, Logical Memory and Paired Associated Words Tests, Token Test, Supra Span Corsi Block Tapping Test, Verbal Fluency Tasks, Raven Colored Matrices, the Rey Complex Figure, Clinical Dementia Rating Scale (CDR) [34], and the Hachinski Ischemic Scale. Thirty-two patients were late and three early AD were onsets; all cases were sporadic. MMSE and CDR scales were used to assess the severity of dementia. AD patients were divided into two subgroups according to disease duration: 17 newly diagnosed AD patients (ADn, disease duration ≤ 2 years) and 18 chronical AD patients (ADc, disease duration > 2 years). The mean MMSE for AD group was 21.2 + 1.8 (scores 6–27).

The diagnosis of MCI was based on the following unanimously adopted criteria: (1) reported cognitive decline; (2) impaired cognitive function; (3) essentially normal functional activities; and (4) exclusion of dementia [35, 36]. The mean age of MCI patients (17 males and 14 females) was 74.2 years (age range 57–82). All of these patients received neurological examination, laboratory test, and brain MRI to exclude intracranial mass, infarcts, moderate to severe nonspecific white matter disease, and reversible causes of cognitive impairment. The same neuropsychological battery discussed in the AD section was used for MCI patients. All patients had a follow-up visit every 6 months, and the monitoring period was 3 years. Based upon subsequent diagnosis status at follow-up evaluations, MCI participants can be divided into two subgroups: 13 MCI patients who have converted to AD (MCI converters, MCIc) and 18 MCI patients who have not converted to AD (MCI nonconverters, MCInc). The mean MMSE for MCI group was 27.6 ± 1.6 (scores 25–29).

Twenty patients with OND (11 females and 9 males, age 59–79 years, mean age 76.4 ± 13.1 years) were also enrolled in the study. These patients with the following conditions: 2 strokes, 5 transient ischemic attacks, 4 chronic intractable headache, 3 status epilepticus, 3 normal pressure hydrocephalus, and 3 peripheral neuropathies. Plasma samples were also obtained from 24 healthy elderly subjects (HC), age and sex matched with the patient (11 males and 10 females, age 57–81 years, mean age 74.7 ± 14.5 years). These individuals were either unrelated healthy spouses of AD and MCI patients or healthy volunteers, and they had no family history of dementia or evidence of acute or chronic diseases at the time of enrollment. The cognitive status of OND and HC was assessed by administration of MMSE (score for inclusion as normal control subjects ≥ 28). All formal neurocognitive test scores for these participants were within 1.5 standard deviations of normative data in published studies or manuals.

Patients with an inflammatory or infectious disease, with a history of immunological or malignant disease, medication of immunologically relevant drugs, abnormal white blood cell count, and abnormal CSF findings, were not included into the study. All study procedures were approved by the Harbin Medical University, China, Institutional Review Board, and all participants or their representatives gave informed consent. All of blood and CSF samples were obtained at the initial visit. In those MCI converters, the second blood and CSF samples were available when they received a diagnosis of AD during followup. 

### 2.2. CSF and Plasma Samples

After lumbar puncture, CSF samples (20–30 mL) were obtained and collected in polypropylene tubes. The samples were centrifuged at 2,000 g at 4°C for 10 minutes to eliminate cells and other insoluble material and were then immediately frozen and stored at −80°C pending biochemical analyses, without being thawed or refrozen. Cell count was performed on the CSF samples and no sample contained more than 500 erythrocytes/*μ*L. Ethylenediamine tetraacetic acid (EDTA) or citrate plasma was obtained by venous puncture. Plasma was isolated by centrifugation and stored at −80°C until use.

### 2.3. Determination of OPN in Plasma and CSF

The OPN protein content in serum and CSF was measured using a commercial ELISA according to the manufacturer's instructions (Assay Designs, Inc., Ann Arbor, MI, USA). Briefly, serum and CSF samples were diluted, respectively, 1 : 20 and 1 : 50 into Assay Buffer provided by the manufacturer and were incubated at 37°C for 1 h in microtiter plates precoated with a polyclonal N-terminal capture anti-OPN antibody (Assay Designs). Then, the plates were washed and wells were incubated at 4°C for 30 min with a horseradish peroxidase labeled OPN-specific monoclonal antibody (Assay Designs). After washing, the wells were incubated with tetramethylbenzidine-H_2_O_2_ solution for 30 min. The color reaction was stopped by adding a solution containing 1 N sulfuric acid. Optical densities were measured at 450 nm with reference wavelength set at 590 nm. The OPN concentrations were calculated using a standard curve of recombinant human OPN provided by the manufacturer. The lower detection limit of the kit was 3.33 ng/mL. Baseline and follow-up CSF and plasma samples from a patient were measured on the same plate. Assays were repeated when the difference between the two probes of one sample was more than 10%.

### 2.4. Statistical Analysis

Normally distributed data sets were analysed by Student's *t*-test, paired *t*-test, analysis of variance (ANOVA), and linear regression and correlation analysis (using “Primer for Biostatistics”). *P* < 0.05 was considered significant.

## 3. Results

### 3.1. OPN Levels in Plasma

The demographic characteristics of the patient material are given in Table 1. There was no difference in sex distribution in MCI, AD, and OND patients and the healthy control subjects.

**Figure 1:**
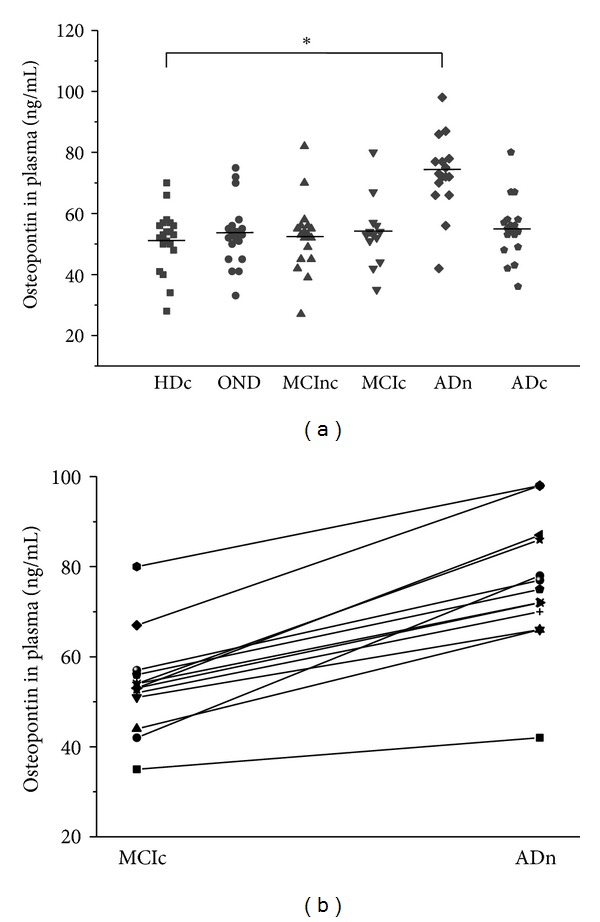
Plasma osteopontin levels in newly diagnosed Alzheimer's disease (AD) are elevated. The horizontal lines represent the mean values of each group, and asterisks show statistical significance (*P < 0.01). (a) Osteopontin concentrations in plasma of 20 healthy control (HC), 20 aged-matched other noninflammatory neurologic diseases (OND), 18 clinically stable mild cognitive impairment patients (MCInc), 13 mild cognitive impairment converters (MCIc), 17 newly diagnosed AD (ADn), and 18 chronical AD patients (ADc) were determined using an enzyme-linked immunosorbent assay (ELISA). (b) The plasma osteopontin concentrations within the same individual (*n* = 13) at different disease statuses.

Plasma OPN concentrations in healthy controls (51.4 ± 9.8 ng/mL) and OND (53.3 ± 10.3 ng/mL) did not differ significantly and did not correlate with age and sex. Plasma OPN concentrations in MCInc (52.3 ± 11.7 ng/mL), MCIc (53.7 ± 11.1 ng/mL), ADn (74.4 ± 13.7 ng/mL), ADc (54.8 ± 10.1 ng/mL), OND patients, and healthy controls differed significantly (Figure 1(a); *P* < 0.001). ADn patients had higher plasma concentrations of OPN than the healthy controls (*P* < 0.005), whereas plasma concentrations of OPN in the MCInc, MCIc, and ADc patients did not differ significantly from plasma OPN concentrations in OND or healthy controls (Figure 1(a)).

We next questioned whether the plasma OPN concentrations would vary within the same individual in relation to disease status. In 13 MCIc individuals tested longitudinally, the plasma OPN concentrations were significantly elevated when they received a diagnosis of AD during followup (Figure 1(b)).

### 3.2. OPN Levels in CSF

The CSF represents the fluid compartment that is closest to reflect the inflammatory situation in the degenerative processes of the nervous system, so we then sought to compare the concentrations of OPN in CSF from patients with MCI, AD, and OND.

**Figure 2:**
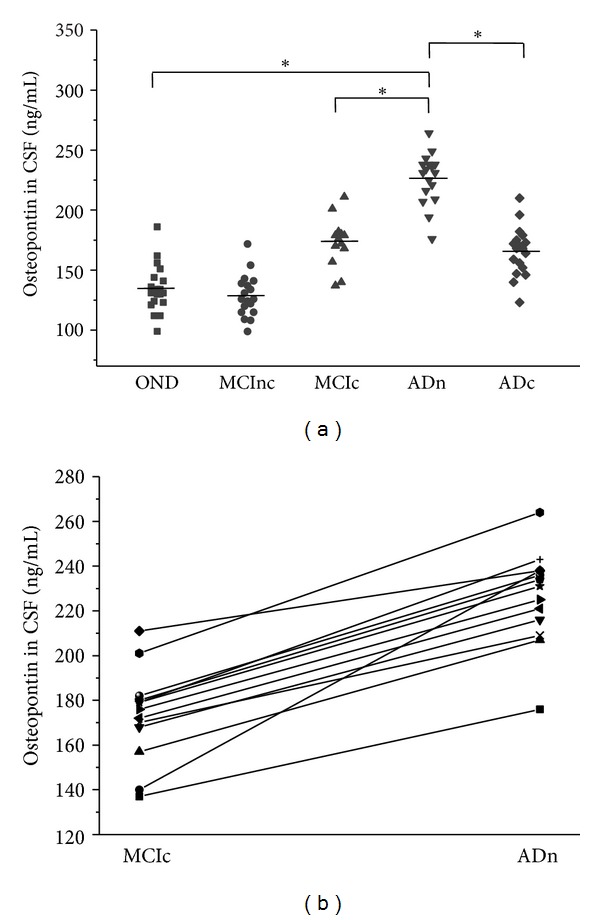
Osteopontin protein levels in the cerebrospinal fluid (CSF) of each group patients. The horizontal lines represent the mean values of each group, and asterisks show statistical significance (*P < 0.01). (a) Osteopontin concentrations in CSF of 20 other noninflammatory neurologic diseases (OND), 18 clinically stable mild cognitive impairment patients (MCInc), 13 mild cognitive impairment converters (MCIc), 17 newly diagnosed AD (ADn), and 18 chronical AD patients (ADc) were determined using an enzyme-linked immunosorbent assay (ELISA). (b) The CSF osteopontin concentrations within the same individual (*n* = 13) at different disease statuses.

CSF OPN concentrations differed significantly in MCInc (128.6 ± 17.7 ng/mL), MCIc (173.2 ± 20.6 ng/mL), ADn (226.5 ± 21.2 ng/mL), ADc (165.6 ± 20.4 ng/mL), and OND patients (134.5 ± 19.3 ng/mL) (Figure 2(a)). Patients with MCIc, ADn, and ADc (*P* < 0.001) had significantly higher OPN concentrations in the CSF than the neurological controls. The concentrations of OPN in CSF from patients with ADn was significantly higher than that from patients with ADc (*P* < 0.001). CSF concentrations of OPN did not differ in MCInc and OND patients.

We also determined whether the CSF OPN concentrations would vary within the same individual in relation to disease status. In 13 MCIc individuals tested longitudinally, the CSF OPN concentrations were significantly elevated when they received a diagnosis of AD during followup (Figure 2(b)).

**Figure 3:**
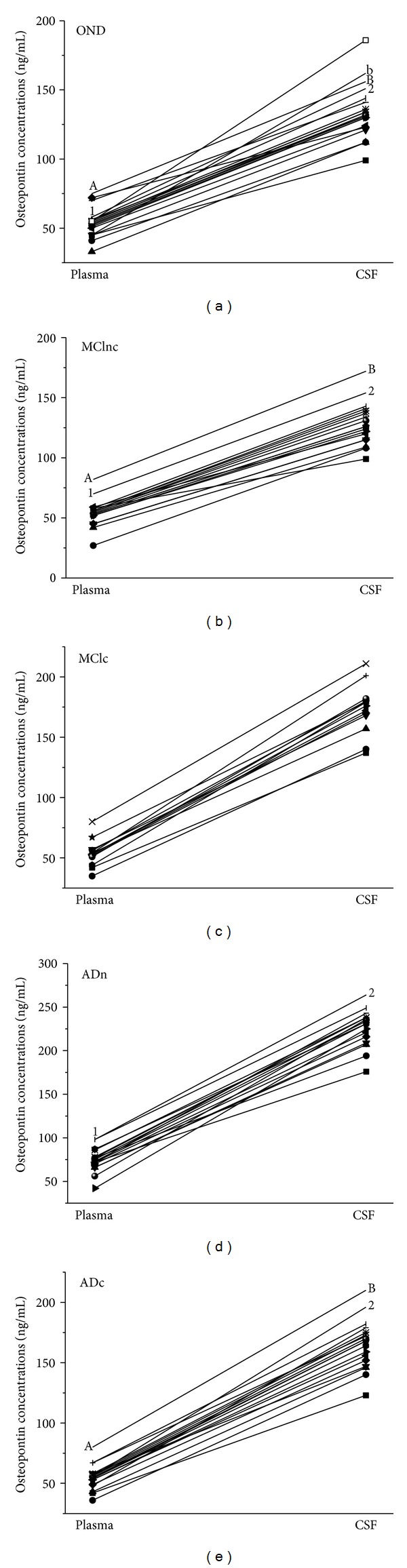
The concentrations of osteopontin in the cerebrospinal fluid (CSF) were significantly higher compared to peripheral blood in patients with other noninflammatory neurologic diseases (OND, *n* = 20), clinically stable mild cognitive impairment patients (MCInc, *n* = 18), mild cognitive impairment converters (MCIc, *n* = 13), newly diagnosed AD (ADn, *n* = 17), and chronical AD patients (ADc, *n* = 18).

When comparing paired CSF and blood samples from patients with MCI, AD, and OND, the concentrations of OPN in the CSF were significantly higher than plasma concentrations in all patients (Figure 3).

### 3.3. Correlation between CSF OPN Concentrations and Clinical Parameters in Patients with AD

**Figure 4:**
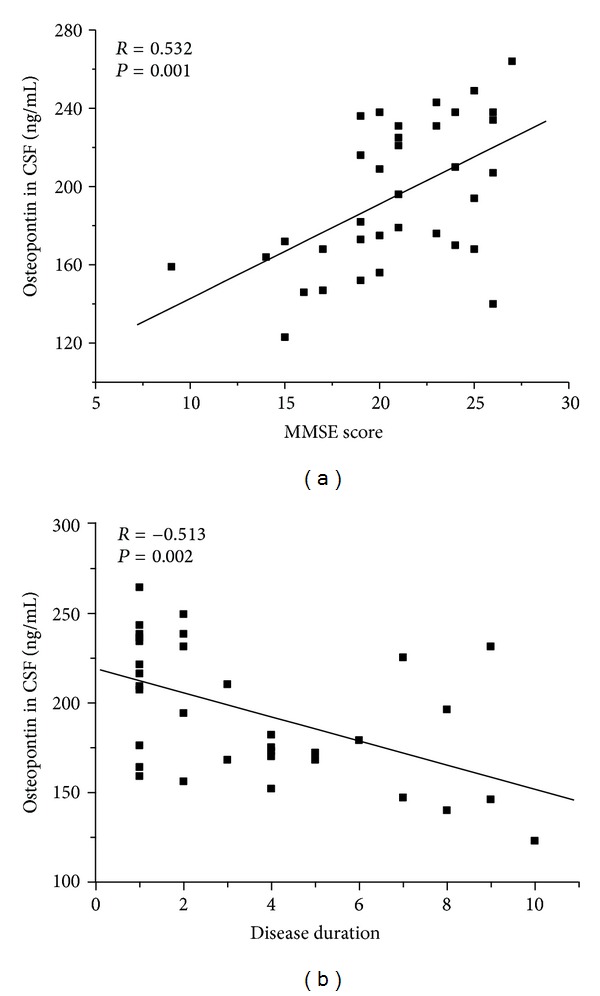
Correlation coefficients were computed to evaluate the relationship between cerebrospinal fluid (CSF) osteopontin levels and the degree of cognitive decline or disease duration in AD patients. (a) A positive correlation between CSF osteopontin levels and the Mini-Mental State Examination Score (MMSE) score in AD patients. (b) The CSF OPN concentration was correlated inversely with the disease duration.

First, we determined whether there was a correlation between the levels of OPN in the CSF and the degree of cognitive decline in AD patients. A strongly positive correlation between the CSF OPN concentrations and the MMSE score (*r* = 0.53, *P* < 0.001) was observed (Figure 4(a)). Secondly, we explored whether there was a correlation between the levels of OPN in the CSF and disease duration of AD patients. Our result showed that the CSF OPN concentration was correlated inversely with the disease duration (*r* = −0.51, *P* < 0.001) (Figure 4(b)).

No other clinical parameters, such as gender, age, A*β*42, tau, and *P* tau levels, had any significant correlation with the levels of OPN in the CSF of AD patients.

Although a trend was noted toward the positive correlation between the plasma OPN concentrations and the MMSE score in the ADn patients, it was not statistically significant.

By contrast, no clear correlation was found between the CSF OPN concentrations of MCIc and both the degree of cognitive decline and disease duration.

## 4. Discussion

In this study, the crosswise comparison demonstrated that CSF OPN concentrations were significantly increased in AD and MCI converters compared to OND, and OPN protein levels both in the CSF and plasma of newly diagnosed AD patients were higher than that of chronical patients. Furthermore, in MCI converters individuals tested longitudinally, both plasma and CSF OPN concentrations were significantly elevated when they received a diagnosis of AD during followup. Finally, OPN CSF levels displayed direct correlation with the MMSE score and inverse correlation with disease duration.

Our data have shown that the plasma concentrations of OPN were significantly increased in the group of newly diagnosed AD than the other groups, and there was a trend toward the positive correlation between the plasma OPN concentrations and the MMSE score, although it was not statistically significant. One previous study on serum OPN concentrations in AD patients gave a different result from ours. Comi et al. found that there were no significant differences in serum OPN concentrations comparing AD to controls [29]. The results of the present study suggest that the differences between the results of the previous study may, at least partly, be explained by that we stratified patients according to their disease duration. Studies demonstrated that high levels of plasma OPN were well correlated with the activities of various disease conditions: plasma OPN concentrations were significantly higher in systemic lupus erythematosus patients and increase in OPN concentration correlated positively and significantly with SLEDAI score in all patients [18]; serum OPN concentrations of patients with idiopathic retroperitoneal fibrosis were elevated compared to healthy controls and correlated with the transverse diameter of the periaortic cuff as determined by imaging studies [37]. In addition, elevated plasma OPN levels have been also shown to play an important role in inflammatory and degenerative processes of the central nervous system: OPN plasma levels were elevated in secondary progressive MS compared to relapsing-remitting MS patients in remission and healthy controls, supporting a role for OPN in the chronic disease activity [38]; OPN serum levels were elevated in Parkinson's disease and higher serum levels were associated with more severe motor symptoms [39]. 

Prospective epidemiological studies show that elevated plasma levels of acute phase reactants can be considered as a risk factor for AD. Therefore, we speculate that elevated OPN plasma levels in the initial stages of AD may contribute to the progression of cognitive decline, although the exact role of OPN and its underlying mechanism as a key proinflammatory cytokine in AD is not understood. Th17 cells have been shown to be a pathogenic effector cell for development of various inflammatory and autoimmune diseases. Shinohara and colleagues found that OPN plays a critical positive role in the differentiation of Th17 cells by repressing IL-27 secretion in mouse dendritic cells [40]. A recent report showed that Th17 T cells were increased in AD patients [41], which favors the speculation that elevated OPN plasma levels in AD may be associated with the differentiation of Th-17 cells.

Accumulating evidence suggests that inflammation mainly occurs in pathologically vulnerable regions of the AD brain (such as the entorhinal, temporoparietal, and cingulate cortex), with increased expression of acute phase proteins and proinflammatory mediators [42]. Therefore, we further examined the concentrations of OPN in the CSF, which directly contact with brain and can accurately reflect the ongoing inflammatory process in the central nervous system. Our results showed that the CSF concentrations of OPN were significantly increased in patients with MCI progressing to AD than that in stable MCI. Prediction of conversion from MCI to AD is of major interest in AD research, which would allow for the appropriate application of disease-modifying treatments at a point where clinical manifestations are limited. Presently, there are few clinical or imaging markers for the early identification of MCI which progresses to AD and MCI which does not progress. Recently, Simonsen and colleagues found that a phosphorylated C-terminal fragment of OPN was increased in the CSF of patients with MCI progressing to AD as compared to patients who remain stable over time and healthy controls and proposed OPN as a biomarker to predict the progression of MCI to overt AD [28]. The findings of these two studies showed that not only the intact forms of OPN but also the cleaved form of OPN was increased in the CSF of patients with MCI progressing to AD. More importantly, our results showed that the CSF OPN concentrations of MCIc were significantly elevated when they received a diagnosis of AD during followup. Our findings are in agreement with another study, which showed that AD patients displayed about a two-fold increased OPN levels in the CSF compared to age-matched controls and it was particularly striking in the early stages of the disease [29]. To identify protein changes during the presymptomatic phase of AD, Ringman and colleagues performed proteomic analyses of CSF from persons with or at risk for inheriting familial AD using high-resolution liquid chromatography-mass spectrometry [43]. Their results showed that OPN was elevated in the CSF of familial AD mutation carriers compared to related noncarriers, which suggest changes of OPN in the CSF occurring a decade before clinical dementia. In addition, increased serum and CSF OPN levels were also detected in the Lewy dementia, and the genotypic variation of SNP-66 was associated with the occurrence of the Lewy body dementia [44]. These findings suggest that OPN is associated with the occurrence of cognitive decline.

Studies showed that OPN seemed to act as a double-edged and might exert two opposite functions in the progression of neurodegenerative diseases. On one hand, OPN functions as a neuroprotectant by upregulating myelination and remyelination. On the other hand, OPN had a disease-accelerating role by triggering neuronal toxicity and death. In the current study, though we could not directly conclude that the increased levels of OPN within the CSF stimulate the degeneration of cerebral neurons and synapses in AD, we conjecture that OPN may favor AD development because MCIc patients had high levels of CSF OPN and the concentrations of OPN in the CSF and plasma were further increased when they received a diagnosis of AD. Our speculation is supported by the study of Wung and collaborators. Their results demonstrated that OPN expression was increased in the pyramidal neurons of the CA1 region of the hippocampus of AD patients and OPN staining intensity positively correlated with both amyloid-beta load and age [27]. Increased OPN expression may exacerbate the abnormal immune response presented in the AD brain by enhancing the survival of activated T cells, which were detected in the brain tissues of AD patients [45].

In conclusion, the bell-shaped curve of CSF OPN expression in disease progression of cognitive decline has extended the evidence for a role of OPN in AD pathogenesis. It will be important to study larger cohorts of individuals with longer durations of followup, and from different centers, to further evaluate whether higher baseline level of CSF OPN was associated with a more marked decline of MMSE over followup.

## Figures and Tables

**Table 1:** Clinical details and laboratory findings of HC, OND, MCI, and AD patients.

	HC	OND	MCInc	MCIc	ADn	ADc
Individuals, no.	20	20	18	13	17	18
Gender, no. of female/male	10/10	11/9	8/10	9/4	11/6	8/10
Mean age (mean ± SD)	72 (57–81)	71 (58–82)	73 (59–83)	72 (60–82)	73 (61–81)	74 (59–83)
MMSE score (mean ± SD)	29 ± 1.5	28 ± 1.7	27.1 ± 1.4	27.9 ± 1.6	22.9 ± 2.7	18.9 ± 4.3
OPN plasma levels (ng/mL) (mean ± SD)	51.4 ± 9.8	53.3 ± 10.3	52.3 ± 11.7	53.7 ± 11.1	74.4±13.7	54.8 ± 10.1
OPN CSF levels (mean ± SD)	—	134.5 ± 19.3	128.6 ± 17.7	173.2 ± 20.6	226.5 ± 21.2	165.6 ± 20.4
A*β* CSF levels (pg/mL) (mean ± SD)	—	326.7 ± 45.8	361.2 ± 48.1	181.7 ± 32.6	167.8 ± 26.9	196.3 ± 29.6
Tau CSF levels (pg/mL) (mean ± SD)	—	112 ± 21.6	141 ± 25.8	243.7 ± 31.6	275.4 ± 34.9	289.6 ± 38.9

HC: healthy control; OND: non-inflammatory neurologic diseases; MCInc: MCI non-converters; MCIc: MCI converters; Adn: newly diagnosed AD; ADc: chronical AD; OPN: osteopontin; SD: standard deviation.
